# Measuring and Quantifying Impacts of Environmental Parameters on Airborne Particulate Matter in Under-Viaducts Spaces in Wuhan, China

**DOI:** 10.3390/ijerph18105197

**Published:** 2021-05-13

**Authors:** Lihua Yin, Tian Hang, Fanfan Qin, Xueting Lin, Yiwen Han

**Affiliations:** 1 Department of Landscape Architecture, School of Architecture and Urban Planning, Huazhong University of Science & Technology, No. 1037 Luoyu Road, Wuhan 430074, China; yinlihua2012@hust.edu.cn (L.Y.); m201973535@hust.edu.cn (T.H.); ifanssy@alumni.hust.edu.cn (F.Q.); m201977292@hust.edu.cn (X.L.); 2 Hubei Engineering and Technology Research Center of Urbanization, No. 1037 Luoyu Road, Wuhan 430074, China; 3 Wuhan Urban Flood Control Survey and Design Institute Co., Ltd., No. 28 Liuhe Road, Wuhan 430014, China

**Keywords:** under-viaducts space, particulate matter, environmental parameters, Wuhan city

## Abstract

Particulate pollution caused by urban traffic emissions has become a significant public hazard. Many urban roads of under-viaduct spaces (UVSs) have become concentrated areas of particulate pollution. This study aims to explore the effects of landscape parameters on particulate matter in UVSs in Wuhan, China. We selected 14 types of UVS sections and nine potential environmental parameters to monitor four types of particulate matter (PM_1.0_, PM_2.5_, PM_10_, and TSP). Finally, linear regression analysis was employed to quantify the relative contributions of environmental parameters to the reduction in the concentration of the four types of particulate matter in the summer and winter. The results showed that particulate matter concentrations exhibit spatial and seasonal differences in UVSs. A single landscape parameter was correlated with particulate matter concentration, while compound environmental parameters had significant effects on the particulate matter concentration in UVSs. Meteorological factors and greening structures had a dominant impact on the particulate matter concentrations in summer and winter, respectively. Therefore, adjusting and optimizing the environmental parameters could reduce particulate pollution in UVSs and could have practical significance for the planning and design of UVSs.

## 1. Introduction

Air pollution caused by rapid urbanization harms the balance of urban ecosystems, the health of residents, and socioeconomic development [1]. Among the various urban air pollutants, particulate matter poses a severe threat to human health; these pollutants can cause lung diseases as well as respiratory and immune system degradation [2,3] and shorten the average human life expectancy by 8.6 months [4].

China has been experiencing severely degraded air quality conditions owing to rapid economic growth, industrialization, and urbanization [5,6]. For example, the number of deaths due to respiratory diseases in Chinese cities (e.g., Beijing, Guangzhou, and Shanghai) increased from 7700 to 8500 from 2010 to 2012 [7]. Although the recent decreasing trend in pollution concentrations has been linked to the effective implementation of emission-reduction strategies from 2015 to 2017 [8,9], air pollution is still vast and multiple challenges await China in the near future.

Road traffic emissions are the primary source of urban particulate pollution [10] and can threaten the health of residents if they experience long-term exposure to busy traffic areas [11]. The construction of road viaducts in high-density urban areas is an efficient way to ease traffic congestion and improve transportation efficiency [12]. Additionally, the use of under-viaduct spaces (UVSs) is essential for the efficient creation of urban spaces. A UVS is defined as the space covered by an orthographic projection below a viaduct and the projection area of the viaduct body created by the local minimum solar incident angle on the winter solstice [13,14]. Current uses of UVSs include parking, greening, and commercial and leisure areas [13,14]. Haydn argued that UVSs can be used as temporary public spaces to address the accessibility characteristics of UVSs in Tokyo, Japan [15]. Xia et al. discussed space design principles and improvement measures for UVS implementation in central Harbin, China [16]. However, UVSs have become areas with increased particulate pollution caused by traffic, owing to their relatively closed environments [12,17]. Therefore, efficiently reducing particulate matter concentrations has been the focus of many studies on the use of UVSs as public spaces.

Urban environmental parameters determine whether particulate matter can be abated, and meteorological factors (especially wind speed) are considered to be essential factors that influence particulate pollution [18]. Additionally, high temperature and low humidity are both conducive to reducing particulate matter concentration [19]. Large green spaces can adsorb more particulate matter to alleviate the negative impact on neighboring residents’ health [20,21]. In contrast, an extremely high-density building can hinder the diffusion of particulate matter, resulting in a higher degree of pollution [22]. In recent years, scholars have begun to pay attention to the ecosystems within UVSs. Optimization of the hydrological regulation and particle dispersion in UVSs can be determined based on environmental parameters, such as meteorological factors, greening structures, and the height and distance of surrounding buildings [23]. However, few studies have been conducted to investigate the effects of these environmental parameters on particulate matter concentrations in UVSs with different viaduct structures.

This study explores the effects of environmental parameters on particulate matter in UVSs. Using Wuhan, China as an example, we selected 14 types of UVS sections and nine potential environmental parameters. Based on the survey and monitoring of four types of typical particulate matter (including particulate matter with an aerodynamic diameter ≤ 10 µm (PM_10_), ≤2.5 µm (PM_2.5_), ≤1.0 µm (PM_1.0_), and total suspended particulates (TSP)), the research focuses on three aspects: (1) the spatial and seasonal distribution characteristics of particulate matter of UVSs in Wuhan, (2) the characteristics of the effects of environmental parameters on particulate matter concentration, and (3) the practical significance of optimizing environmental parameters for the abatement of particulate matter in UVSs.

## 2. Methodology

Considering the diversity of viaducts and greening structure types in Wuhan’s UVSs, 14 UVS sections were selected as samples for monitoring in this study. Based on satellite images and field investigations, the temperature, humidity, and concentrations of particulate matter (PM_1.0_, PM_2.5_, PM_10_, and TSP) were measured with temperature-humidity recorders and dust monitors. We explored the effects of nine environmental parameters of UVSs on the four particulate matter types through statistical analyses.

### 2.1. Study Area and Measurement Sites

**Figure 1:**
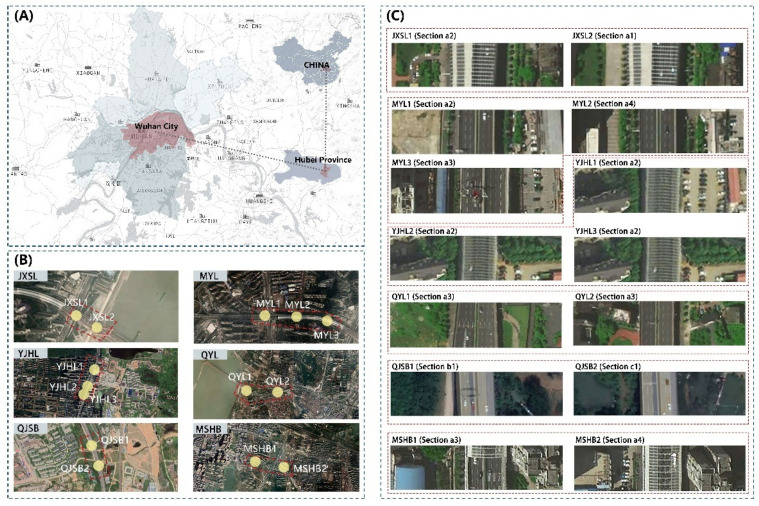
The (**A**) study area and location distribution of 14 UVS sections, (**B**) satellite images, and (**C**) cross-section combinations.

The city of Wuhan (30°60′ N, 114°30′ E), a metropolis in central China (Figure 1A), is dominated by the northern subtropical humid monsoon climate with an extremely hot summer, cold winter, and abundant rainfall. Its annual average temperature is 28 °C, and the annual average precipitation ranges from 770 mm to 1570 mm. Over the past decade, the particulate pollution in Wuhan has become increasingly prominent, and road dust is among the primary sources of pollution [24]. The viaduct, being a crucial part of Wuhan’s transport system, produces most of the particulate pollution. By August 2016, 43 viaducts had been constructed in Wuhan with a total length of 120.5 km. Simultaneously, the UVSs of Wuhan covered a total area of approximately 220.77 hm^2^ [13]. According to China’s National Ambient Air Quality Standard (NAAQS), the standard values of the annual average PM_10_ and PM_2.5_ concentrations for Level 2 air quality are 70 μg/m^3^ and 35 μg/m^3^, respectively. In 2013, the compliance rates of PM_10_ and PM_2.5_ concentrations in Wuhan were 69.6% and 51.5%, and the annual average PM_10_ and PM_2.5_ concentrations were 124 μg/m^3^ and 94 μg/m^3^, respectively. Until 2018, the compliance rates of PM_10_ and PM_2.5_ concentrations of Wuhan were 96.3% and 85.1%, and the annual average PM_10_ and PM_2.5_ concentrations were 73 μg/m^3^ and 49 μg/m^3^, respectively. These values exhibited a decline of 41.1% and 47.9%, respectively, compared to the 2013 values, but failed to meet the NAAQS for Level 2 air quality. Therefore, particulate pollution remains severe in Wuhan.

### 2.2. Data Collection

#### 2.2.1. Sampling Site Selection

**Figure 2:**
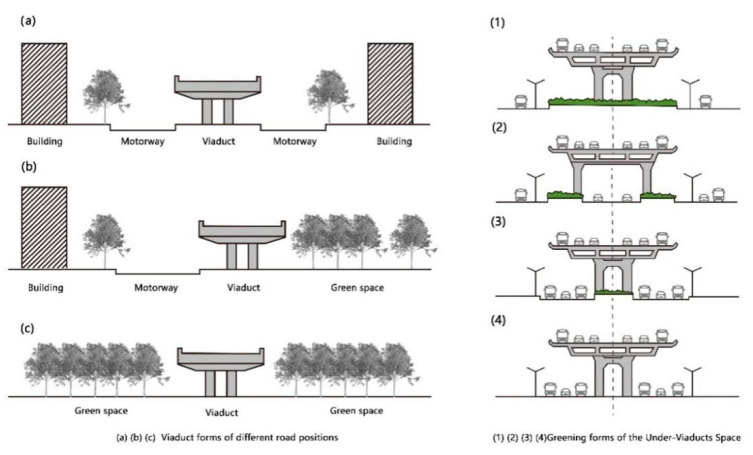
(**a**–**c**) viaduct structure in Wuhan and (**1**)–(**4**) UVS greening patterns. The sample combination form is selected as a1, a2, a3, a4, b1, and c1.

Both the location and quantity of pollution sources affect particulate matter concentration [25]. The primary source of particulate matter in UVSs is vehicle emissions [26]. We selected UVS sample sites based on the following criteria: (1) the sampling viaducts were located in central urban areas of Wuhan; (2) the UVS sample sites were selected from the two-way urban main roads with six lanes, considering a specific difference in traffic volume and particulate pollution between roads of different hierarchies; and (3) the section types comprised of viaduct structures (Figure 2a–c) and greening patterns of UVSs (Figure 2(1)–(4)) that were not repeated after they were combined. Based on these criteria, 14 UVS sections were selected as sample sites for further monitoring and modeling (Figure 1B,C).

#### 2.2.2. Measuring Particulate Matter Concentration

The monitoring period selected was during typical summer and winter periods in Wuhan. Moreover, the concentrations of PM_1.0_, PM_2.5_, PM_10_, and TSP were measured on sunny and windless or breezy days (wind speed ≤ 5.4 m/s) to avoid the potential impact of meteorological factors (e.g., cloud, precipitation, strong wind, and severe air pollution). Additionally, to reduce the measuring error between sampling sites, the particulate matter concentration was measured during peak traffic hours, namely, from 07:00 to 09:00 and 17:00 to 19:00 (Beijing time). A complete monitoring period comprised of three consecutive days and three periods were conducted during each season. The mean concentration during the monitoring periods was used as the final concentration.

**Figure 3:**
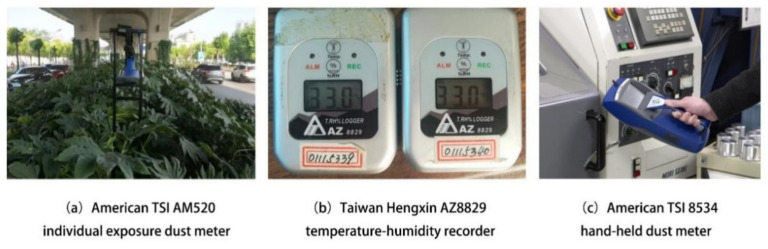
Experimental monitoring instruments.

The particle measurement of 14 UVS samples included moving-point and fixed-point monitoring simultaneously [25]. The moving-point measurement data are used as the actual measurement data of the UVSs, whereas the fixed-point measurement data are used as reference data for the external background of the viaduct. At the fixed-point outside each viaduct, we set up one individual exposure dust meter (TSI AM520, TSI Incorporated, Shoreview, MN, USA) (Figure 3a) and one temperature-humidity recorder (Hengxin AZ8829, AZ, Taizhong, Taiwan) (Figure 3b) for the fixed-point measurements. Fixed-point measurement data were recorded at regular 10 s intervals. The PM_1.0_, PM_2.5_, PM_10_, and TSP concentrations were monitored during three-day periods. All fixed-point monitors were set in the center of the sidewalk outside the viaduct (3–10 m away from the viaduct) and at 1.5 m above the ground [27,28]. The moving-point data were measured using a hand-held dust meter (TSI 8534, TSI Incorporated, Shoreview, MN, USA) (Figure 3c) and a temperature-humidity recorder (Hengxin AZ8829, AZ, Taizhong, Taiwan) (Figure 3b), which were cyclically moved between the measuring points of the fixed-point detection on the UVS centerline. The measurement data were read and recorded at regular 10 s intervals, and the PM_1.0_, PM_2.5_, PM_10_, and TSP concentrations were monitored simultaneously. Three consecutive groups of measured values were selected for calculating the mean particulate matter concentration at the moving-point. Finally, owing to a deficiency in instruments, we were only able to acquire size data for one type of particulate matter for fixed-point monitoring each day. The calculated values according to PM_10_/TSP [29,30] and PM_2.5_/PM_1.0_ [31,32] ratios were used as alternatives.

Considering the potential spatial background difference of airborne particle level in the urban environment, we took the net concentration difference (dTSP, dPM_10_, dPM_2.5_, and dPM_1.0_) between the moving points and corresponding fixed points as indicators of the difference in particle concentration at each sampling site [25]. A positive concentration difference in particles indicated that the concentration of particles in the UVSs exceeded that outside the UVSs and vice versa. The data were calculated as follows:

dPM_1.0_ = CPM_1.0_sp − CPM_1.0_ck (1)

dPM_2.5_ = CPM_2.5_sp − CPM_2.5_ck (2)

dPM_10_ = CPM_10_sp − CPM_10_ck (3)

dTSP = CTSPsp − CTSPck(4)

where d denotes the net concentration difference between the four types of particulate matter; C denotes the concentration of different types of particulate matter; sp denotes moving-point monitoring; and ck denotes fixed-point monitoring. According to the above equations, the net temperature difference (dTa) and net humidity difference (dRH) can be determined.

#### 2.2.3. Measuring and Calculating Greening and Environmental Parameters in UVSs

According to the field investigation results, we formulated the following screening criteria for environmental parameters: (1) based on previous studies, potential environmental parameters affecting the particulate matter concentration were identified [19,22,33]; (2) the indicators of selected environmental parameters were quantifiable; and (3) the extracted environmental parameters were all available in the 14 UVS samples. Finally, nine potential environmental parameters affecting the particulate matter concentration in the UVS were selected and analyzed quantitatively (Table 1).

The detailed procedure included the following steps: (1) the temperature and humidity (Ta and RH, respectively) in the UVSs were measured by the temperature-humidity recorder; (2) the greenbelt width (UVSgw) and greening area (UVSga) in the UVSs were measured using a measuring tape and laser range finder, and the UVSga was calculated; (3) the ambient greening area (Aga) around the UVS sample sites, ambient building distance (Abd), ambient building area (Aba) (i.e., the rectangular range of 50 m distances from the outer sides of each viaduct body), and ambient opening degree (Aod) (i.e., the proportion of the space not blocked by buildings or trees at the vertical height of >1.5 m within the study range) were calculated based on satellite images and field measurement data; and 4) the viaduct height (H) and viaduct breadth (B) were determined from the laser range finder and satellite images, respectively, and the breadth-height ratio (B/H) was calculated.

### 2.3. Data Analysis

After acquiring the monitoring data, we comparatively analyzed the dPM_1.0_, dPM_2.5_, dPM_10_, and dTSP in the UVS sample sites using one-way analysis of variance. We further explored the relationship between the concentration difference of the four particulate matter types and the nine environmental parameters through linear regression analysis [25]. Then, the multiple stepwise regression (backward) model was used to quantify the relative contributions made by environmental parameters and the reductions in the TSP, PM_10_, PM_2.5_, and PM_1_ concentrations in the summer and winter. The prediction model is as follows:

Y = a + b_1_X_1_ + b_2_X_2_ + b_3_X_3_ + ... + b*_n_*X*_n_* (*n* ≤ 9) (5)

where Y denotes CPM_1.0_, CPM_2.5_, CPM_10_, or CTSP; a is a constant; b_1_, b_2_, ... b_n_ denote the coefficients of independent variables; and X_1_, X_2_, ... X*_n_* are the landscape element parameters. The statistical analysis process was performed using SPSS 22.0 (IBM, Armonk, NY, USA). For the regression analysis results, a *p* value of <0.05 was considered to be statistically significant.

## 3. Results

### 3.1. Characteristics of Particulate Matter Concentrations in UVSs

**Figure 4:**
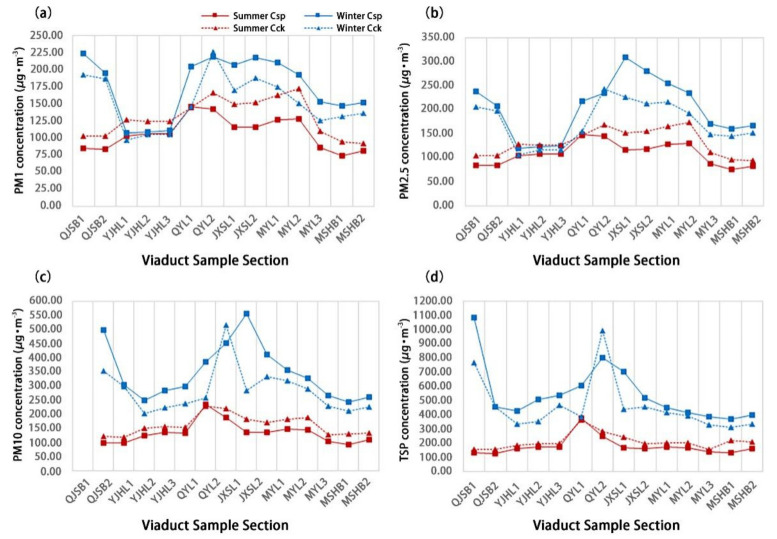
The general trend of particulate matter concentration in 14 UVS sections in summer and winter. (**a**) PM_1_ concentration, (**b**) PM_2.5_ concentration, (**c**) PM_10_ concentration, and (**d**) TSP concentration.

As shown in Figure 4, the particulate matter concentration in UVSs exhibited significant spatial variation characteristics. At the 14 sample sites, the mean concentrations of the four types of particulate matter in the summer differed significantly from those in the winter. Additionally, the mean concentration in winter was higher than in summer. Furthermore, the particulate matter was distributed evenly in summer but varied significantly in winter. In the summer, the moving-point concentration of the four types of particulate matter reached the peak in the QYL1 section, where the moving-point and fixed-point concentrations were very close to each other. In contrast, all the moving-point concentrations were lower than the fixed-point concentrations at the other 13 monitoring points. In the winter, the moving-point concentrations of PM_1.0_ and TSP reached the peak in the QJSB1 section, whereas those of PM_2.5_ and PM_10_ reached the peak in the JXSL1 section. Excluding the QYL2 section, these concentrations of the four particulate matter types exceeded the fixed-point concentrations at the other 13 monitoring points.

**Figure 5:**
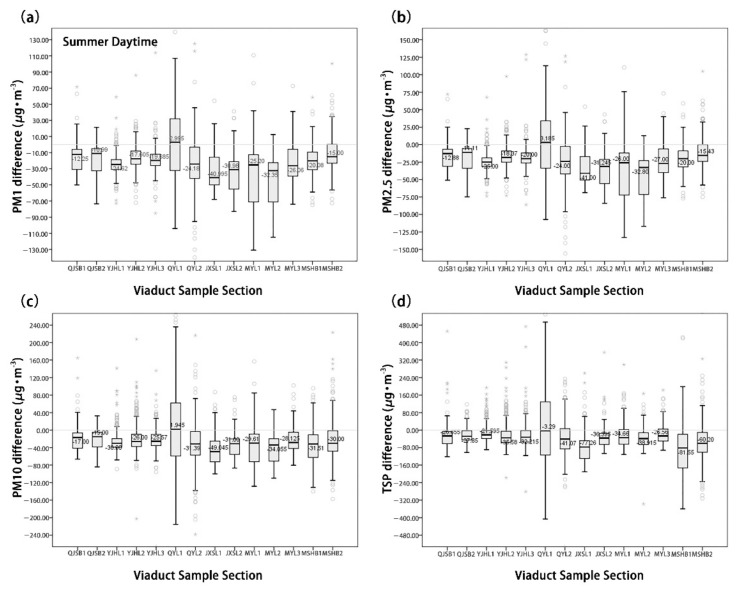
Comparison of the concentration difference of four types of particulate matter in the summer. (**a**) PM_1_ concentration difference, (**b**) PM_2.5_ concentration difference, (**c**) PM_10_ concentration difference, and (**d**) TSP concentration difference.

**Figure 6:**
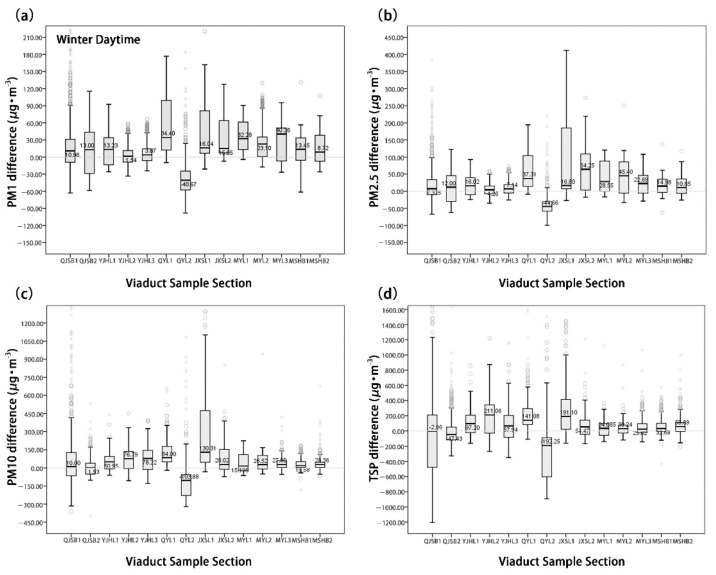
Comparison of the concentration difference of four types of particulate matter in the winter. (**a**) PM_1_ concentration difference, (**b**) PM_2.5_ concentration difference, (**c**) PM_10_ concentration difference, and (**d**) TSP concentration difference.

Figure 5 and Figure 6 show that the moving-point concentrations of 14 UVS samples are significantly different in summer and winter. The moving-point concentration of particulate matter in the UVS was generally lower than the fixed-point particulate matter concentration outside the viaducts in the summer. However, it was higher than the fixed-point particulate matter concentration outside the viaducts in the winter. Additionally, the absolute value of the net concentration difference across the four types of particulate matter was larger in winter, indicating that the particulate matter concentration in the UVS varied more significantly in winter than in summer.

In the summer, the mean concentration of the fine particulate matter (PM_1.0_, PM_2.5_) dPM_1.0_ was −22.92 μg/m^3^ and the mean concentration of dPM_2.5_ was −23.24 μg/m^3^. Except in the QYL1 section, their mean concentrations at the 13 other sample sites were negative. The mean concentration of the coarse particulate matter (PM_10_, TSP) dPM_10_ was −26.51 μg/m^3^, and the mean concentration of dTSP was −33.33 μg/m^3^; the maximum concentrations of both types appeared in the QYL1 section. In the QYL1 section, the concentration difference in coarse particulate matter was maximized and exhibited the most significant variation.

In the winter, the mean concentration of fine particulate matter dPM_1.0_ was 19.34 μg/m^3^, while fine particulate matter dPM_2.5_ was 27.11 μg/m^3^. Except in the QYL2 section, their mean concentrations at the other 13 sites were positive. The mean concentrations of coarse particulate matters dPM_10_ and dTSP were 62.77 μg/m^3^ and 85.78 μg/m^3^, respectively. Except in the QYL2 section, the mean concentrations of dPM_10_ at the other 13 sites were positive. Except in the QYL2 and QJSB2 sections, the mean concentrations of dTSP at the other 12 sites were positive.

### 3.2. Effects of Environmental Parameters on Airborne Particulate Matter

**Figure 7:**
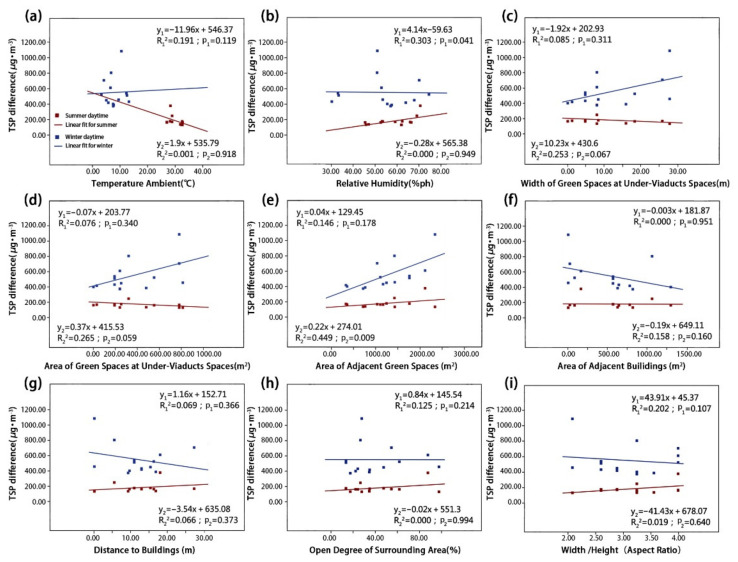
The relationships between TSP concentration difference and (**a**) TA, (**b**) RH, (**c**) UVSgw, (**d**) UVSga, (**e**) Aga, (**f**) Aba, (**g**) Abd, (**h**) Aod and (**i**) B/H in the summer and winter.

**Figure 8:**
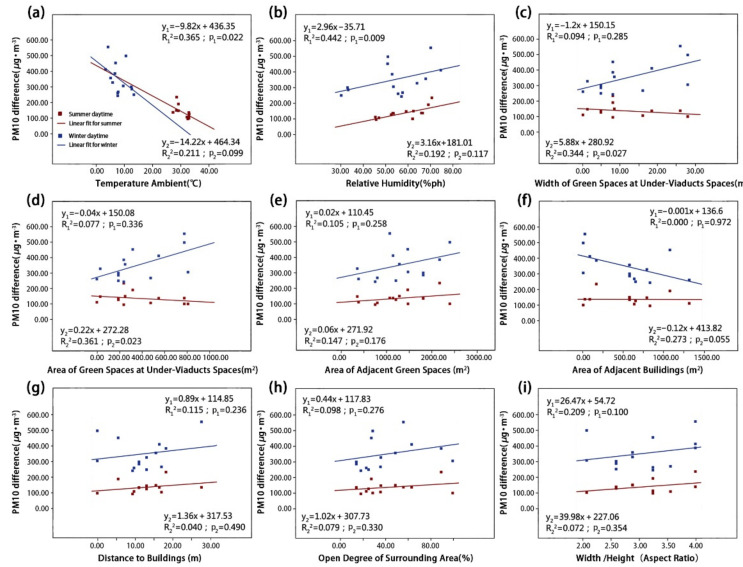
The relationships between PM_10_ concentration difference and (**a**) TA, (**b**) RH, (**c**) UVSgw, (**d**) UVSga, (**e**) Aga, (**f**) Aba, (**g**) Abd, (**h**) Aod and (**i**) B/H in the summer and winter.

**Figure 9:**
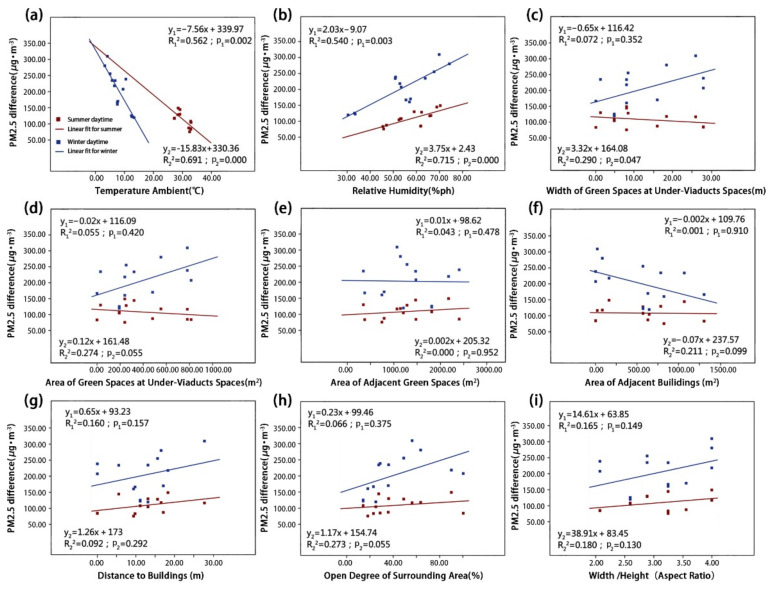
The relationships between PM_2.5_ concentration difference and (**a**) TA, (**b**) RH, (**c**) UVSgw, (**d**) UVSga, (**e**) Aga, (**f**) Aba, (**g**) Abd, (**h**) Aod and (**i**) B/H in the summer and winter.

**Figure 10:**
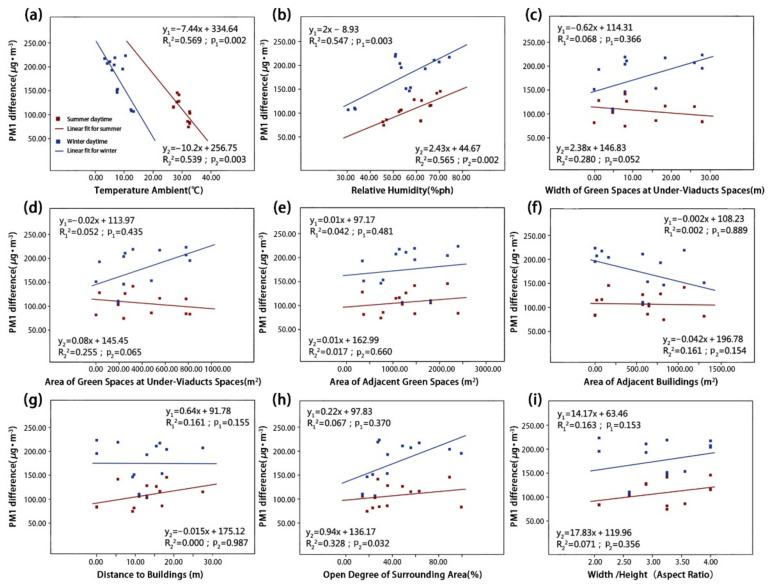
The relationships between PM_1.0_ concentration difference and (**a**) TA, (**b**) RH, (**c**) UVSgw, (**d**) UVSga, (**e**) Aga, (**f**) Aba, (**g**) Abd, (**h**) Aod and (**i**) B/H in the summer and winter.

We used a linear regression model to visualize the influence of different environmental parameters on particle reductions. These reductions are represented by the net concentration differences of the four types of particulate matter in the summer and winter (Figure 7, Figure 8, Figure 9 and Figure 10). The correlation degree is represented by the coefficient of determination (R^2^).

#### 3.2.1. Effects of Environmental Parameters on a Single Type of Particulate Matter

As shown in Figure 7 and Figure 8, the concentrations of coarse particulate matter dTSP and dPM_10_ were both correlated with RH and Aga. Simultaneously, the dPM_10_ concentration was also correlated with Ta and UVSgw. First, the dTSP concentration was positively correlated with RH (Figure 7b; p_1_ = 0.041) in the summer, and it was found that the concentration difference of dTSP decreases with increasing RH. In winter, the dTSP concentration had no significant correlation with RH, but had a strong positive correlation with Aga (Figure 7e; p_2_ = 0.009) (note: dTSP did not have a significant correlation with Aga in the summer). Furthermore, the dTSP concentration had no significant correlation with other environmental parameters in the summer or winter. Second, the dPM_10_ concentration was positively correlated with Ta (Figure 8a; p_1_ = 0.022) and RH (Figure 8b; p_1_ = 0.009) in the summer, but there was no significant correlation in the winter. The dPM_10_ concentration was positively correlated with UVSgw (Figure 8c; p_2_ = 0.028) and UVSga (Figure 8d; p_2_ = 0.023) in the winter, but there was no significant correlation in the summer. dPM_10_ exhibited no significant correlation with other environmental parameters in summer or winter.

As shown in Figure 9 and Figure 10, dPM_2.5_ and dPM_1.0_ were both significantly correlated with Ta and RH, respectively, and dPM_2.5_ and dPM_1.0_ were correlated with UVSgw and Aod, respectively. The dPM_2.5_ concentration had a very significant negative correlation with Ta in both summer and winter (Figure 9a; p_1_ = 0.002; p_2_ = 0.000), but showed a significant positive correlation with RH (Figure 9b; p_1_ = 0.003; p_2_ = 0.000). The dPM_2.5_ concentration was positively correlated with UVSgw in the winter, but exhibited no significant correlation in the summer (Figure 9c; p_2_ = 0.047). Other environmental parameters were not significantly correlated with the dPM_2.5_ concentration. In both summer and winter, the dPM_1.0_ concentration was significantly negatively correlated with Ta (Figure 10a; p_1_ = 0.002; p_2_ = 0.003), but was significantly positively correlated with RH (Figure 10b; p_1_ = 0.003; p_2_ = 0.002). Additionally, the dPM_1.0_ concentration was positively correlated with the Aod in the winter (Figure 10h; p_2_ = 0.032). Other environmental parameters were not significantly correlated with the dPM_1.0_ concentration.

#### 3.2.2. Correlation Degree of Different Environmental Parameters on Particulate Matter

Table 2 shows the degree of determination coefficients of the concentration differences of the four types of particulate matters and nine environmental parameters. In total, 16 correlations in the summer were higher than those in the winter, whereas 20 correlations in the winter were higher than those in the summer. Evidently, environmental parameters in the winter affected the concentration difference of particulate matter in the UVS more significantly than in the summer, which may be related to poor air quality caused by increased heating and fossil fuel combustion in winter [25,34]. The concentration difference of fine particulate matter was significantly correlated with Ta and RH. The impact of greening elements on particulate matter was second only to Ta and RH. The impact of Aga on the concentration difference of coarse particulate matter significantly exceeded that of fine particulate matter, and the impact of dTSP reached a significance level of 0.05.

### 3.3. Multiple Regression Predictive Models of Environmental Parameters and Net Concentration Differences of Particulate Matter

Through a multiple linear regression model (backward method), we investigated the effect of different combinations of environmental parameters in the summer and winter on the net concentration difference of the four types of particulate matter in the UVS. The coefficient of determination (R^2^) refers to the change ratio of the net concentration difference of each type of particulate matter, as explained by the predictive regression model. The standardization coefficient (beta coefficient) refers to the relative contribution of different environmental parameters to the change in the net concentration difference of particulate matter.

#### 3.3.1. Summer Prediction Model

First, in summer, approximately 91.0% of the variation in dTSP was jointly explained by four environmental parameters (dTSP = −1711.484 + 33.256 Ta + 11.453 RH − 0.166 UVSga + 87.910 B/H) (Table 3). The dTSP concentration was most significantly affected by RH, followed by Ta, with beta coefficients of 1.525 and 1.214, respectively. A 10% increase in RH would increase the dTSP concentration by 114.53 μg/m^3^ in the summer. Second, approximately 96.0% of the variation in dPM_10_ was jointly explained by six environmental parameters (dPM_10_ = − 128.353 + 2.788 RH − 0.055 UVSga + 0.035 Aga + 0.058 Aba + 1.098 Abd + 0.523 Aod). The dPM_10_ concentration was most significantly affected by Aba, and its beta coefficient was 0.630. A 10% increase in Aba would increase the dPM_10_ concentration by 0.58 μg/m^3^ in the summer. Finally, approximately 95.9% of the variation in dPM_2.5_ and dPM_1.0_ in the summer was jointly explained by three environmental parameters. In the dPM_2.5_ model, the dPM_2.5_ concentration was most significantly affected by RH, followed by UVSgw with beta coefficients of 0.957 and −0.626, respectively. According to the dPM_2.5_ model, a 10% increase in RH would increase the dPM_2.5_ concentration by 26.44 μg/m^3^ in the summer (dPM_2.5_ = −35.385 + 2.644 RH − 1.513 UVSgw + 0.358 Abd). In the dPM_1.0_ model, the dPM_1.0_ concentration was most significantly affected by RH, followed by UVSgw with beta coefficients of 0.958 and −0.619, respectively. A 10% increase in RH would increase the dPM_2.5_ concentration by 25.90 μg/m^3^ in the summer (dPM_1.0_ = −34.467 + 2.590 RH − 1.463 UVSgw + 0.353 Abd).

#### 3.3.2. Winter Prediction Model

First, approximately 92.8% of the variation in dTSP in the winter was jointly explained by six environmental parameters (dTSP = 9636.803 − 408.430 Ta − 93.070 RH + 213.995 UVSgw − 6.212 UVSga − 6.816 Aod − 262.021 B/H) (Table 4). The dTSP concentration was most significantly affected by UVSgw, and its beta coefficient was 10.528. A 10% increase in UVSgw would increase the dTSP concentration by 2139.95 μg/m^3^. Second, approximately 94.1% of the variation in dPM_10_ in the winter was jointly explained by seven environmental parameters (dPM_10_ = 4415.145 − 190.056 Ta − 39.637 RH + 77.014 UVSgw − 2.127 UVSga + 3.228 Abd − 2.524 Aod − 170.174 B/H). The dPM_10_ concentration was most significantly affected by UVSgw, and its beta coefficient was 7.681. A 10% increase in UVSgw would increase the dTSP concentration by 770.14 μg/m^3^.

In the PM_2.5_ regression model, approximately 96.9% of the variation in dPM_2.5_ in the winter was jointly explained by five environmental parameters (excluding UVSga and Aod from the seven environmental parameters) (dPM_2.5_ = 1527.777 − 67.602 Ta − 11.031 RH + 21.708 UVSgw − 0.585 UVSga + 1.370 Abd − 0.480 Aod − 80.255 B/H). In the winter, the dPM_2.5_ concentration was most significantly affected by Ta, followed by UVSgw with beta coefficients of −3.550 and 3.522, respectively. A 10% increase in Ta would reduce the dPM_2.5_ concentration by 676.02 μg/m^3^. In the dPM_1.0_ regression model, approximately 94.4% of the variation in dPM_1.0_ in the winter was jointly explained by five environmental parameters (dPM_1.0_ = 1252.466 − 53.590 Ta − 9.374 RH + 19.967 UVSgw − 0.585 UVSga − 54.632 B/H). The dPM_1.0_ concentration was most significantly affected by UVSgw, and its beta coefficient was 4.442. According to the dPM_1.0_ regression model, a 10% increase in UVSgw would increase the dPM_1.0_ concentration by 199.67 μg/m^3^.

## 4. Discussion

Motor vehicle emissions are one of the primary sources of atmospheric particulate pollution in urban areas [10,35,36]. The particulate concentration distribution in the urban road environment generally exceeds that in the adjacent urban environment. UVS is a unique urban road traffic space. A decrease in particulate pollution is necessary for developing and using UVSs comprehensively and reasonably. Based on field monitoring and regression modeling, this study explored the relationship between environmental parameters and particulate matter concentrations in UVSs. The results provide a theoretical foundation for the abatement of particulate matter in UVSs and improvement of the utilization value of UVSs.

### 4.1. Differences in Particulate Matter Concentration in Urban UVSs

The distribution of particulate matter concentration in urban environments is characterized by spatial and seasonal variations [18]. The results from this study show that the concentrations of four particulate matter types in UVSs were lower than those in the adjacent road environment in the summer but higher than those in the adjacent road environment in the winter. This may be related to the unique structure of UVSs, as the diffusion of airborne particulate matter is affected by aerodynamic mechanisms [37]. In summer, the temperature and humidity in UVSs are lower and higher, respectively, than those outside the bridge. Differences in temperature and humidity may result in different patterns of the air flow, facilitating a reduction in particulate matter in UVSs. Contrarily, in winter, air flow can promote an increase in particulate matter in UVSs. Moreover, the viaduct structure above UVSs hinders the vertical diffusion of some particulate matter [38,39]. We found that the higher the B/H ratio, the lower the particulate matter concentration (Table 3 and Table 4). This is due to vegetation in UVSs, which plays a positive role in abating particulate matter [40,41]. The result also proves that high Aod is beneficial to particulate matter diffusion and that higher Aod values decrease particulate matter concentration [22,33]. In summer, the net particulate matter concentration difference was directly proportional to Abd and Aod.

The concentration of urban airborne particulate matter exhibited a strong correlation with meteorological factors characterized by seasonal variation [32,42,43,44]. The net concentration difference across four particulate matter types in UVSs was significantly greater in the winter than in the summer. This is because, in the winter, there is less green deciduous vegetation and urban air quality is generally worse [25,34,45]. The differences between the particulate matter concentration in UVSs and adjacent road environments were relatively stable in the summer but fluctuated significantly in the winter. This is attributable to the influence of climatic characteristics. In fact, under winter meteorological conditions, such as static temperature and high humidity, the original secondary ions in the air are easily converted into PM particles [45,46].

### 4.2. Effects of Environmental Parameters on Particulate Matter Concentration

Different environmental parameters had varying degrees of effects on particulate matter concentrations. Correlation analysis showed that a single landscape element had relatively slight effects on the particulate matter concentration in UVSs (Table 2). However, regression analysis revealed that multiple environmental parameters collectively affected the particulate matter concentration difference in UVSs significantly (Table 3 and Table 4). Specifically, meteorological factors (e.g., Ta and RH) and greening structures (e.g., VSgw and VSga) significantly affected the particulate matter concentration in UVSs (Table 3 and Table 4). First, high air temperature was beneficial for reducing particulate matter concentration. An increase in temperature not only accelerates the transmission and diffusion of particulate matter by increasing the frequency of vertical air convection, but also strengthens the photosynthesis and adsorption of particulate matter by plants [42,47,48]. Generally, as RH increased, the concentration of particulate matter also increased in urban spaces [19,49,50]. The regression model showed that RH was positively correlated with the concentration difference of particulate matter in the summer but negatively in the winter. This indicates that, when RH increases to a certain level, the increase in wet deposition is beneficial for reducing particulate matter concentration [42].

Second, greening structures in UVSs can affect the particulate matter concentration in UVSs. The larger the greening area is, the more significant the absorption and adsorption effects are [21]. Additionally, the greening density significantly affected the particulate matter concentration in UVSs. When greening was extremely sparse, the retention and blocking effects of vegetation on particulate matter were relatively poor. Contrarily, when greening was extremely dense, the significant blocking effect increased the particulate matter concentration in UVSs [51,52,53,54]. Therefore, reasonable greening of UVSs facilitates air permeation and diffusion [41].

### 4.3. Practical Significance

Urban air pollution poses serious environmental health risks and severe threats to humans and ecosystems [55,56]. Particulate pollution from road traffic, in particular, is becoming increasingly severe [57]. Furthermore, with the large-scale construction of urban viaducts and rapid increase in the number of UVS areas [14,16], environmental health should be used as an indicator for measuring the development and utilization of UVSs. First, the orientation and structure design of urban viaducts should consider the effects of meteorological factors on particulate matter [58,59]. Urban viaducts along the direction of ventilation corridors are beneficial for abating particulate matter around the viaduct body [60]. The B/H ratio of viaducts can affect particulate matter diffusion in UVSs [23]. Second, optimization of the built environment around UVSs plays a positive role in reducing the particulate matter concentration. Abd and Aod are the main reference conditions for converting UVSs into urban public spaces [22,33,61] (Table 3 and Table 4). Finally, UVSs can be managed on a time-sequence basis because of the seasonal differences in particulate matter concentrations in urban road environments. For example, the particulate matter concentration in UVSs is extremely high in winter, and therefore, people are advised to avoid gathering activities below urban viaducts. During the initial stages of urban viaduct construction, environmental health and safety and the functions of UVSs should be considered strategically to achieve efficient use of land in high-density urban areas.

Optimization of greening structures is an essential measure for improving the environment of UVSs. Excessive greening density in UVSs will hinder particulate matter diffusion [41] and, therefore, it is necessary to consider the specific landscape characteristics of UVSs comprehensively. First, large and medium-sized trees should be managed to prevent them from hindering particulate matter diffusion [51,52,53,54]. Second, an optimal scrub height can be determined according to the planting height of scrubs in street canyons [62,63], thus effectively abating particulate matter in UVSs.

## 5. Conclusions

This study shows that the concentrations of particulate matter of different sizes exhibit spatial and seasonal differences in the UVSs of Wuhan. The mean concentrations of four different types of particulate matter in the winter were significantly higher than those in the summer. Mean concentrations in UVSs were lower than those in adjacent areas in the summer but higher than those in adjacent areas in the winter. Only some of environmental parameters exhibited a minor individual correlation with the particulate matter concentration in UVSs. In contrast, the collective effects of the compound environment, comprising of nine environmental parameters, on the particulate matter concentration in UVSs were significant. In the summer, meteorological factors had dominant effects on the particulate matter concentration in UVSs, while in the winter, greening structures had dominant effects on the particulate matter concentration in UVSs. Additionally, the viaduct structure and ambient built environment can affect the particulate matter concentration in UVSs. Therefore, adjusting and optimizing environmental parameters is essential for reducing particulate pollution in UVSs and has practical significance to the planning of urban viaducts and the design and utilization of UVSs.

This study had several limitations owing to constraints related to the number of monitoring samples and measuring instruments. First, the selected sample sites covered the common UVS types observed in Wuhan; however, obtaining accurate regression analysis results requires more sample sites to be monitored. Second, owing to the uncertainty in the microenvironment and data availability regarding particulate matter concentrations and the environment, the interpretation of statistical data contains some inaccuracy. The restrictions imposed by the complexity of the spatiotemporal scale on the scientific interpretation of the correlations between environmental parameters and particulate matter concentrations should also be verified. Further studies should focus on the effects of environmental parameters on the abatement of particulate matter in UVSs and determine a theoretical basis for the sustainable utilization of UVSs.

## Figures and Tables

**Table 1:** The environmental parameters of the 14 UVSs samples.

Viaduct Sample Sections	Ta		RH		UVSgw	UVSga	Aga	Aba	Abd	Aod	B/H
	Summer	Winter	Summer	Winter							
	(°C)	(°C)	(%ph)	(%ph)	(m)	(m^2^)	(m^2^)	(m^2^)	(m)	(%)	
QJSB1	32.64	10.46	61.81	50.88	25	780	2398.48	0.00	0	28.72	2.08
QJSB2	32.59	9.50	61.73	53.31	26	813	1468.83	0.00	0	99.48	2.08
YJHL1	32.71	13.25	52.37	30.32	4.8	192	1199.51	647.94	26	25.06	2.6
YJHL2	32.58	12.53	53.02	33.39	4.8	192	1807.98	573.66	22	14.23	2.6
YJHL3	32.60	12.35	53.32	33.08	4.8	192	1807.98	573.66	22	14.23	2.6
QYL1	28.31	6.57	70.26	52.74	8	240	2171.00	162.13	36.25	89.34	3.56
QYL2	28.92	6.80	68.73	50.75	8	320	1463.02	1073.27	11	27.52	2.89
JXSL1	26.99	4.16	65.68	69.77	26	780	1063.49	0	55	55.90	4
JXSL2	27.04	3.26	66.19	74.19	18.4	552	1136.72	81.25	32.74	63.18	4
MYL1	28.20	5.06	62.13	67.57	8.4	252	1278.00	572.80	30.91	48.77	2.6
MYL2	28.90	5.78	58.93	63.72	1.2	30	342.00	787.71	26	35.93	2.6
MYL3	31.91	7.73	46.94	57.34	16	480	786.79	632.27	33.88	35.63	3.2
MSHB1	32.22	7.57	45.71	56.88	8	240	739.52	826.53	18.6	18.22	2.89
MSHB2	32.62	7.53	45.36	55.38	0	0	370.00	1312.86	19.36	23.05	2.89

Note: Ta, RH, UVSgw, UVSga, Aga, Aba, Abd, Aod, and B/H refer to the ambient temperature, relative humidity, width of green spaces in UVSs, area of green spaces in UVSs, area of adjacent green spaces, area of adjacent buildings, distance to buildings, open degree of the surrounding area, and width/height ratio (aspect ratio), respectively.

**Table 2:** Determination coefficients of the four-sized particle concentration differences and nine environmental parameters.

	dPM_1.0_		dPM_2.5_		dPM_10_		dTSP	
	R_1_^2^	R_2_^2^	R_1_^2^	R_2_^2^	R_1_^2^	R_2_^2^	R_1_^2^	R_2_^2^
Ta	**0.569**	**0.539**	**0.562**	**0.691**	**0.365**	0.211	0.191	0.001
RH	**0.509**	**0.565**	**0.540**	**0.715**	**0.442**	0.192	**0.303**	0.000
UVSgw	0.068	0.280	0.072	**0.290**	0.094	**0.344**	0.085	0.253
UVSga	0.052	0.255	0.055	0.274	0.077	**0.361**	0.076	0.265
Aga	0.042	0.017	0.043	0.000	0.105	0.147	0.146	**0.449**
Aba	0.002	0.161	0.001	0.211	0.000	0.273	0.000	0.158
Abd	0.161	0.000	0.160	0.092	0.115	0.040	0.069	0.066
Aod	0.067	**0.328**	0.066	0.273	0.098	0.079	0.125	0.000
B/H	0.163	0.071	0.165	0.180	0.209	0.072	0.202	0.019

Note: R_1_^2^ refers to the determination coefficients in summer, and R_2_^2^ to those in winter. Boldface represents significant variables with *p* < 0.05.

**Table 3:** Regression analysis of the four-sized particle concentration differences and nine environmental parameters in the summer.

Variables	Summer dPM_1.0_			Summer dPM_2.5_			Summer dPM_10_			Summer dTSP		
	Coefficient		Sig. ^c^	Coefficient		Sig. ^c^	Coefficient		Sig. ^c^	Coefficient		Sig. ^c^
	B ^a^	Beta ^b^		B ^a^	Beta ^b^		B ^a^	Beta ^b^		B ^a^	Beta ^b^	
Ta										33.256	1.214	**0.001**
RH	2.590	0.958	**0.000**	2.644	0.957	**0.000**	2.788	0.626	**0.001**	11.453	1.525	**0.000**
UVSgw	−1.463	−0.619	**0.000**	−1.513	−0.626	**0.000**						
UVSga							−0.055	−0.393	**0.027**	−0.166	−0.709	**0.000**
Aga							0.035	0.566	**0.003**			
Aba							0.058	0.630	**0.015**			
Abd	0.353	0.221	**0.007**	0.358	0.219	**0.007**	1.098	0.417	**0.004**			
Aod							0.523	0.370	**0.021**			
B/H										87.910	0.900	**0.000**
Constant	−34.467			−35.385			−128.353			−1711.484		
R^2^		0.959			0.959			0.960			0.910	
Adjusted R^2^		0.947			0.947			0.925			0.870	

Note: B ^a^ = unstandardized coefficient; Beta ^b^ = standardized coefficient; Sig. ^c^ = significance level. Boldface represents significant variables with *p* < 0.05.

**Table 4:** Regression analysis of the four-sized particle concentration differences and nine environmental parameters in winter.

Variables	Winter dPM_1.0_			Winter dPM_2.5_			Winter dPM_10_			Winter dTSP		
	Coefficient		Sig. ^c^	Coefficient		Sig. ^c^	Coefficient		Sig. ^c^	Coefficient		Sig. ^c^
	B ^a^	Beta ^b^		B ^a^	Beta ^b^		B ^a^	Beta ^b^		B ^a^	Beta ^b^	
Ta	−53.590	−3.859	**0.000**	−67.602	−3.550	**0.002**	−190.056	−6.136	**0.001**	−408.430	−6.504	**0.000**
RH	−9.374	−2.899	**0.001**	−11.031	−2.488	**0.009**	−39.637	−5.496	**0.001**	−93.070	−6.365	**0.000**
UVSgw	19.967	4.442	**0.010**	21.708	3.522	**0.042**	77.014	7.681	**0.006**	213.995	10.528	**0.001**
UVSga	−0.585	−3.648	**0.019**	−0.585	−2.661	0.077	−2.127	−5.945	**0.013**	−6.212	−8.563	**0.001**
Aga												
Aba												
Abd				1.370	0.329	**0.038**	3.228	0.477	**0.031**			
Aod				−0.480	−0.215	0.098	−2.524	−0.695	**0.004**	−6.816	−0.925	**0.000**
B/H	−54.632	−0.818	**0.001**	−80.255	−0.876	**0.004**	−170.174	−1.142	**0.005**	−262.021	−0.867	**0.003**
Constant	1252.466			1527.777			4415.145			9636.803		
R^2^		0.944			0.969			0.941			0.928	
Adjusted R^2^		0.908			0.932			0.873			0.867	

Note: B ^a^ = unstandardized coefficient; Beta ^b^ = standardized coefficient; Sig. ^c^ = significance level. Boldface represents significant variables with *p* < 0.05.

## Data Availability

Data are contained within the article.
